# Dynamics and interactions of Quincke roller clusters: From orbits and flips to excited states

**DOI:** 10.1126/sciadv.adf5144

**Published:** 2023-05-17

**Authors:** Abraham Mauleon-Amieva, Michael P. Allen, Tanniemola B. Liverpool, C. Patrick Royall

**Affiliations:** ^1^ H.H. Wills Physics Laboratory, Tyndall Avenue, Bristol BS8 1TL, UK.; ^2^ School of Chemistry, University of Bristol, Cantock’s Close, Bristol BS8 1TS, UK.; ^3^ Centre for Nanoscience and Quantum Information, Tyndall Avenue, Bristol BS8 1FD, UK.; ^4^ Bristol Centre for Functional Nanomaterials, Tyndall Avenue, Bristol BS8 1FD, UK.; ^5^ Department of Physics, University of Warwick, Coventry CV4 7AL, UK.; ^6^ School of Mathematics, University of Bristol, Fry Building, Woodland Road, Bristol BS8 1UG UK.; ^7^ Gulliver UMR CNRS 7083, ESPCI Paris, Université PSL, 75005 Paris, France.

## Abstract

Active matter systems may be characterized by the conversion of energy into active motion, e.g., the self-propulsion of microorganisms. Artificial active colloids form models that exhibit essential properties of more complex biological systems but are amenable to laboratory experiments. While most experimental models consist of spheres, active particles of different shapes are less understood. Furthermore, interactions between these anisotropic active colloids are even less explored. Here, we investigate the motion of active colloidal clusters and the interactions between them. We focus on self-assembled dumbbells and trimers powered by an external dc electric field. For dumbbells, we observe an activity-dependent behavior of spinning, circular, and orbital motions. Moreover, collisions between dumbbells lead to the hierarchical self-assembly of tetramers and hexamers, both of which form rotational excited states. On the other hand, trimers exhibit flipping motion that leads to trajectories reminiscent of a honeycomb lattice.

## Teaser

From orbits and flips to electrohydrodynamical coupling and supracolloidal chemistry in active colloidal clusters.

## INTRODUCTION

In recent years, much effort has been devoted to investigating the motility of microorganisms, driven, e.g., by flagellae and to the realization of synthetic active matter by means of diffusophoretic (*1*), thermophoretic (*2*), and field-driven (*3*) active particles. These active particles exhibit fascinating collective phenomena not found in passive systems, such as flock formation (*3*), dynamical clustering and phase separation (*4*–*6*), and anomalous density fluctuations (*7*). While many experiments have focused on self-propelled spherical colloids, e.g., Janus particles, biological microswimmers are often anisotropic (*6*).

Many artificial swimmers display a persistent random walk dominated by ballistic runs and rotational diffusion, whereas the locomotion of microorganisms allows adjustments in their trajectories (*8*). Some synthetic particles with motility akin to that of certain biological agents have been produced, such as an artificial flagellum (*9*); rod-shaped (*10*), ellipsoidal (*11*) and chiral particles (*12*) that exhibit flocking (*13*); asymmetric particles (*14*) that feature gravitaxis (*15*), along with in situ feedback of model vision cones (*16*), i.e., active control (*17*). Another form of active particles that break symmetry through their motility are spinners (*18*), which exhibit rotating crystals (*19*), turbulence (*20*), and exotic phenomena such as odd viscosity (*21*).

In active colloidal systems, attention has often focused on assembly of large numbers of particles, i.e., the emergence of macroscopic states or active “phase behavior” (*3*). By contrast, assembly of fixed numbers of passive colloids, often through careful control of interactions (*22*), has led to supracolloidal chemistry with reaction pathways at the colloidal rather than molecular level (*23*–*27*), which exhibit some aspects of molecular interactions (*28*). However, passive colloids exhibit overdamped dynamics, so collisions as such are very different to those that would occur in atomic and molecular systems (*29*). While many active colloidal systems are diffusive at long times, the persistence length of their motion can be many particle diameters, and, thus, one may inquire as to collisions between active colloids. Collisions between active colloids may thus present some similarity to collisions between atoms and molecules. In the case of “wet” active matter, when active particles come close together, hydrodynamic coupling can lead to the formation of bound states (*30*, *31*) as observed in the volvox algae (*32*), which may be analogous to electronically coupled excited bound states in spectroscopy (*29*).

In experiments, assembly of active clusters has been investigated (*33*, *34*), along with self–assembled spinners (*20*). In simulation, predictions have been made for assembly (*35*) and demixing (*35*, *36*) of small clusters of active colloids (*36*). Cluster assembly of active dipolar particles has been shown to exhibit an unusual fission phenomenon (*37*). Mixtures of anisotropic active particles exhibit even more complex behavior including microphase separation [which can also be seen in some experiments with active monomers (*38*)], fluidization, and three-phase coexistence (*39*). Recently, experiments have been conducted, which explore the interactions between anisotropic active colloids such as spinning microtori (*40*) and chiral clusters (*41*), along with rotational states formed via collisions of upright active disks (*42*). Collective behavior of pear-shaped particles has been investigated (*43*), driven via the Quincke electrorotation of particles (*3*). Interactions have even been manipulated in systems of dumbbells (*44*) and anisotropic “patchy” active colloids (*34*, *45*).

Here, we present an experimental study on the motion of active colloidal dimers and trimers formed from Quincke rollers. These display a characteristic behavior distinct from the dynamics of spherical active particles, particularly circular and jumping motions. The dimers share some characteristics with the pear-shaped Quincke rollers investigated previously (*43*). However, just as in passive matter, the behavior of anisotropic particles is profoundly influenced by aspect ratio [for example the formation of liquid crystalline phases (*46*)], our dumbbells have an aspect ratio close to unity, where dimer crystals form in the case of passive systems (*47*), unlike the finite micellar-like structures formed by pear-shaped particles (*48*).

## RESULTS

Our active colloidal clusters are prepared by taking advantage of attractive interactions between the constituent spheres leading to irreversible binding. Briefly, we use polystyrene beads of diameter σ = 3.1 μm with a polydispersity of 5%. The initial suspension is aqueous, and the colloids are electrostatically stabilized. To remove ionic stabilizing layers, the particles are washed and transferred to a liquid of low conductivity. This leads to the formation of clusters of different sizes. Smaller particles are separated from the bigger ones using centrifugation, and the final suspension is a mixture of single spheres, dumbbells, and trimers (see Materials and Methods).

To investigate the active colloidal clusters, we exploit the Quincke roller mechanism. Particles are confined between two conductive glass slides 30 μm apart. A dc electric field *E* is applied perpendicular to the substrate, leading to the spontaneous symmetry breaking of the charge distribution at the particle-liquid interface. As a result, rotation at a constant rate emerges from an imposed electric torque acting on the particle. For a rigid sphere near to a substrate, the rotation of the particles is coupled with the translation, giving rise to self-propelled rollers, where the speed *v* is controlled by the electric field *E* (*3*). Quincke rotation occurs above a certain threshold field strength *E*_Q_, and this is the regime in which we operate.

We focus on active colloidal dumbbells and trimers. A sequence of dynamic transitions is observed for dumbbells from local spinning to disordered orbital (DO) and then ordered orbital (OO) motions as the activity increases. As a result, dumbbells exhibit an increased trajectory radius and a change of their effective translation and rotational motion. In agreement with the description of a Brownian circle swimmer (*49*), the self-propulsion direction does not strictly coincide with the dumbbell orientation, resulting in circular trajectories. Rather than the collective behavior of a large (unspecified) number of particles, here, we focus on single particles and interactions between two and three particles. When two dumbbells collide, we sometimes observe the formation of an excited state of tetramers that spin quickly. A more complex formation of hexamers formed from a dumbbell colliding with a tetramer is also observed. This excited state turns out to be unstable. We find that the spinning motion of tetramers and hexamers is activity dependent with a coupling between self-propulsion and arrested motion due to steric frustration. We rationalize our observation of the motion and coupling of dumbbells by considering hydrodynamic interactions. Furthermore, while dumbbells and the excited states that result from their collisions roll along the substrate, trimers cannot do so, because of their triangular shape. In this case, we observe an interesting combination of in-plane diffusion and out-of-plane “flipping” motion. This corresponds to a jump-diffusion process that evolves the position and orientation of the trimer discontinuously.

### Dumbbells: Spinning, disordered, and ordered orbiting

**Fig. 1.**
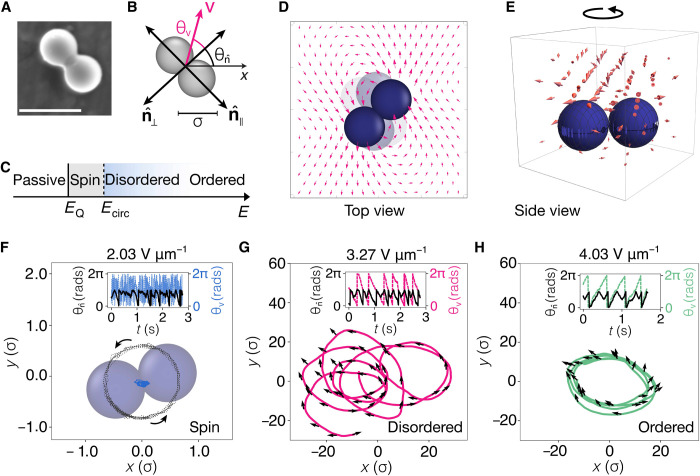
Spinning, DO, and OO motions of dumbbells. (**A**) Scanning electron microscopy (SEM) micrograph of a Quincke dumbbell. Scale bar, 5 μm. (**B**) Representation of a dumbbell body frame. Perpendicular ⊥ and longitudinal ∥ orientations $\hat{n}$ with respect to the bond between the two spheres are shown. In addition, the velocity **v** is given by the displacement of the center of mass **r**. The angles θ*_i_*, corresponding either to the velocity or to the orientation, are defined with respect to the reference axis. (**C**) Field-dependent behavior of dumbbells. Passive dumbbells become active spinners above *E*_Q_ and then circular rollers above *E*_dis_. (**D** and **E**) Schematics of incoming electrohydrodynamic flow field as a result of the field application, whose angular velocity leads to rotation in the horizontal plane (D) and elevated side view (E). Flow fields are obtained with eq. S2. (**F** to **H**) Representative trajectories of the three states. (F) A spinning dumbbell at low *E*. Solid blue line represents the displacement of the center of mass **r**, and the dashed line is from the motion of one of the sites as the dumbbell spins. (G) DO motion and (H) OO motion. Solid lines indicate the displacement of the center of mass, and arrows correspond to the orientation ${\hat{n}}_{⊥}$. Insets in (F) to (H) show the time evolution of the angles θ_**v**_ (dashed lines) and $\theta_{\hat{n}}$ (solid lines).

We start by describing the active motion of Quincke dumbbells. These are elongated rigid particles with a transverse (⊥) and a longitudinal (∥) orientation $\hat{n}=(\cos\theta_{\hat{n}},\sin\theta_{\hat{n}})$, where θ is the angle formed with respect to a reference axis. Figure 1B depicts ${\hat{n}}_{⊥}$ and ${\hat{n}}_{∥}$ with respect to the bond connecting the two spheres. In addition, an angle θ_**v**_ is given for the displacement. For the motion, we apply a range of field strengths *E* ∈ {2,4} V μm^−1^. For low values of *E*, i.e., *E* < *E*_Q_ with *E*_Q_ ≈ 2 V μm^−1^, we obtain passive dumbbells. Above *E*_Q_, a spinning behavior (S) is observed with a constant rate and without a notable displacement of the center of mass **r**, as shown in Fig. 1F. This is distinct from the spinning behavior of pear-shaped rollers found at higher field strengths (*43*, *50*). Here, the onset of the DO motion occurs at higher values of the applied field than that at which the spinning occurs, i.e., *E*_dis_ ≈ 2.5 V μm^−1^. Last, at still higher field strengths, the circular motion becomes localized around a central point, giving rise to OO motion (see Fig. 1F and movie S1). Upon decreasing the field strength, we find that the dumbbells exhibit the same dynamic behavior, that is to say that the sequence S, DO, and OO is reversible.

We rationalize the dynamical behavior of Quincke dumbbells as follows. We start by considering the low–field strength, spinning behavior, before developing an analysis of the orbital motion. In a system of Quincke monomers, at relatively weak field strengths around the critical field strength *E*_Q_ where we find the spinning, there is an electrohydrodynamic flow (*38*, *51*, *52*). This flow (in plane) is toward the dumbbell, with the solvent escaping out of plane as illustrated schematically in Fig. 1 (D and E). In the case of a single dumbbell, angular momentum of the incoming flow then generates a torque, leading to spinning (see the analysis in the Supplementary Materials).

At higher field strengths, the friction due to coupling between the dumbbell and the substrate leads to rolling and a large change in the dynamics. The spinning at low field strengths gives way to an orbital motion when combined with the rolling (Fig. 1, G and H). In our system, there is, furthermore, Brownian translational and rotational diffusion, which may cause some fluctuation in the successive orbits. At relatively low field strengths, this leads to a DO state (Fig. 1G), but it appears to be insignificant at higher field strengths where we find an OO state (Fig. 1H).

We now analyze the DO and OO motions. The dynamics are governed by the self-propulsion velocity *v* and self-spinning angular velocity ω that are taken to be independent

$$\begin{array}{c} \dot{r}=v\hat{n}+\xi ;\\ \dot{\theta }=\omega +\eta \end{array}$$ (1)

where $\hat{n}=(\cos\theta ,\sin\theta )$ and 〈ξ*_i_*(*t*)ξ*_j_*(*t*′)〉 = 2*D*_t_δ*_ij_*δ(*t* − *t*′) and 〈η(*t*)η(*t*′)〉 = 2*D*_r_δ(*t* − *t*′) are variances for the **ξ** and η noise terms.

For the DO and OO trajectories, we find that the dumbbell displacement occurs with a direction θ_**v**_ close to the transverse orientation ${\hat{n}}_{⊥}$, However, these trajectories are a result of the decoupling between the self-propulsion **v** and the dumbbell orientation ${\hat{n}}_{⊥}$, as indicated by the arrows and insets in Fig. 1 (F to H). Here, the direction of motion, i.e., clockwise (+) or anticlockwise (−), is not predefined as in chiral particles (*14*), and, thus, the circular motion is presumably due to torque that arises from the spinning mechanism illustrated in Fig. 1 (D and E).

Following (*49*), the dynamics of noninteracting circle swimmers in two dimensions are given by the overdamped Langevin equations

$$\begin{array}{c} \dot{r}=\beta D\cdot [F\hat{n}+\zeta ],\\ \dot{\theta }=\beta D_{r}[T+\zeta_{\theta }] \end{array}$$ (2)

where β = (*k*_B_*T*)^−1^ is the thermal energy and $F\hat{n}$ is an effective internal force representing the self-propulsion. $D=D_{⊥}(I-\hat{n}⊗\hat{n})+D_{∥}(\hat{n}⊗\hat{n})$ is the dumbbell diffusion tensor, where *D*_⊥_ and *D*_∥_ are the transverse and longitudinal translational diffusion coefficients and **I** is the unit tensor. The rotational dynamics are given by *D*_r_, the rotational diffusion coefficient, and T, the effective torque promoting the circular motion on dumbbells. Last, Gaussian noise terms **ζ** and ζ_θ_ for the displacement and the orientation are added respectively. We observe an enhanced translation during DO and OO trajectories. On the other hand, the rotational motion is diffusive, and angular diffusion coefficients are obtained as (*53*)

$$D_{r}=\langle {\left[{\Delta \theta }_{⊥}(t)\right]}^{2}\rangle /(2t)$$ (3)

Given the equations of motion (Eqs. 1 and 2), we can construct the mean squared displacement MSD = ⟨[**r**(*t*) − **r**(0)]^2^⟩

$$\begin{array}{rl} \mathrm{MSD} & =2v^{2}\{\frac{D_{r}t}{\omega^{2}+D_{r}^{2}}+\frac{e^{-D_{r}t}\cos(\omega t)-1}{\omega^{2}+D_{r}^{2}}-\frac{2D_{r}\omega e^{-D_{r}t}\sin(\omega t)}{{\left(\omega^{2}+D_{r}^{2}\right)}^{2}}\\ & \;-\frac{2\omega^{2}[e^{-D_{r}t}\cos(\omega t)-1]}{{\left(\omega^{2}+D_{r}^{2}\right)}^{2}}\}+4D_{t} \end{array}$$ (4)

The terms proportional to *v*^2^ measure the contributions of orientational correlation to the MSD, while the translational diffusion of the center of mass is proportional to *D*_t_. The spin is driven by ω, and the curvature of the orbital trajectories is due to the interplay between ballistic motion due to *v*, loss of orientational correlations due to rotational diffusion *D*_r_, and spinning due to ω.

**Fig. 2.**
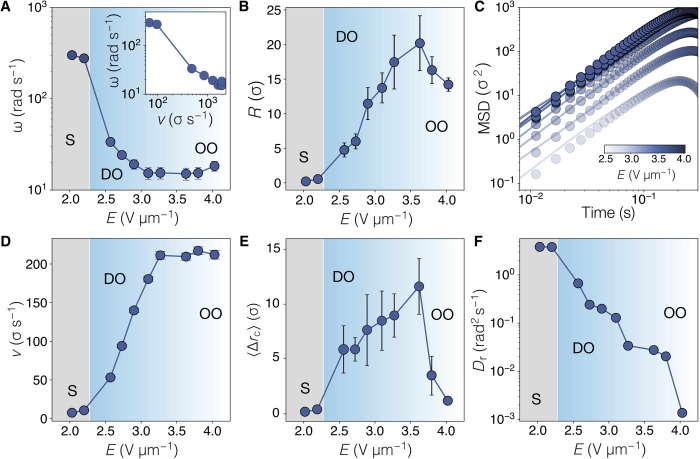
Dynamics of dumbbells. (**A**) Angular velocity ω as a function of the field strength *E*. Two regimes are identified: spin motion (S; shaded region) appears with low values of *E*, whereas DO and OO orbital trajectories emerge with increased *E*. (**B**) Trajectory radius *R* for the different regimes obtained with *E*. (**C**) MSDs measured at different amplitudes of *E*. Symbols are from experiments and solid lines are fits to Eq. 4. (**D**) Self-propulsion speed of dumbbells versus the field amplitude. (**E**) Mean displacement of the trajectory central point **r**_c_. (**F**) Rotational diffusion coefficient *D*_r_ obtained from fits in (C) using Eq. 4.

For a spinning dumbbell, $\frac{v^{2}}{D_{r}}\ll D_{t}$ and ω ≤ *D*_r_, the MSD reduces to ∼4*D*_t_. Equation 4 is recovered for a dumbbell exhibiting DO motion where $\frac{v^{2}}{D_{r}}\gg D_{t}$ and ω ≤ *D*_r_. Last, for the OO trajectories, $\mathrm{MSD}∼\frac{2v^{2}}{\omega^{2}}[1-\cos(\omega t)]$, with $\frac{v^{2}}{D_{r}}\gg D_{t}$ and ω ≫ *D*_r_. MSDs in Fig. 2C are fitted to obtain *D*_r_ from Eq. 4 using the experimentally measured self-propulsion *v* and rotational ω velocities of dumbbells. The extracted values of *D*_r_ are shown in Fig. 2F.

### Coupling of drive and slipping leads to a nonmonotonic angular velocity

Figure 2A shows the relation of the angular velocity ω = ∣Δθ∣/*t* with the applied field *E*. Upon increasing the field strength, we observe a nonmonotonic response of the angular velocity ω of a decay and then an increase. In an opposite manner, the self-propulsion speed *v* increases with the field until it reaches steadiness at *E* > 3 V μm^−1^ (Fig. 2D). Both the increase in *v* and the decrease in ω have an impact on the orbit radius *R* = *v*/∣ω∣. In Fig. 2B, we observe a nonmonotonic behavior, with a peak in *R* at *E* ≈ 3.6 V μm^−1^. The spread in the radii of the orbits is presumably related to the polydispersity of the two particles comprising each dumbbell, as we believe that their different sizes contribute to the orbital motion.

We can find the center of every cycle θ_v_ ∈ {0,2π}, which corresponds to one revolution. To do so, we take the center of the path of the dumbbell around one cycle, which is defined as the point at which direction of motion of the dumbbell is identical to that at the start or end of the previous cycle. The center of each cycle is termed **r**_c_. Each trajectory lasts around *t*_traj_ = 3.5 s. We take the displacement of the center of each cycle, considered between the start and end of the trajectory Δ**r**_c_ = ∣**r**_c_(*t*_traj_) − **r**_c_(0)∣. Δ**r**_c_ is shown in Fig. 2E, which shows the disordered nature of the orbital motion compared to steady orbits at higher *E* (Fig. 2E). Note that this motion is rather smaller but not totally negligible on the time and length scales of the MSD in Fig. 2C).

This behavior contrasts with the rather steady radius in trajectories of asymmetric active particles (*14*). There, the angular velocity ω increases linearly with the speed *v*, while *R* shows a nondependent behavior on the self propulsion. Here, on the other hand, the dependence of ω and *R* on *E* is opposite to the observations of active spheres in a viscoelastic medium (*54*). Thus, our findings suggest that the emerging circular behavior of Quincke dumbbells is due to an effective internal torque and to the field-dependent speed *v*.

**Fig. 3.**
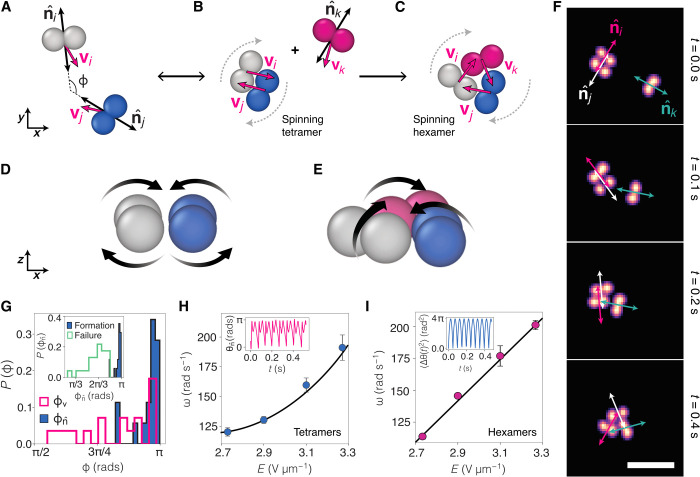
Formation of tetramers and hexamers. (**A**) Active dumbbells performing DO motion may collide with a consequent change in their trajectory. We take the angle ϕ made between the orientations ${\hat{n}}_{ij}$ and velocities **v***_ij_* to characterize the collisions. (**B**) When aligned, two colliding dumbbells form spinning tetramers whose motion results from the dynamical frustration exerted by one dumbbell on the other. (**C**) The formation of hexamers is possible when a third dumbbell collides with a previously formed tetramer. The resulting spinning motion of hexamers is also attributed to the dynamical frustration of single circular trajectories. (**D** and **E**) Schematic representation of the Quincke rotation of dumbbells. Hydrodynamic coupling is schematically illustrated with the black arrows. (**F**) Formation sequence of a hexamer. A tetramer is previously formed by two dumbbells. A third dumbbell approaches with its orientation ${\hat{n}}_{k}$ pointing toward the tetramer. Upon collision, the dumbbells rearrange to form a triangular shape as indicated by the orientations ${\hat{n}}_{ijk}$. Scale bar, 10 μm. (**G**) For dumbbells forming tetramers, the distributions of ϕ indicate that the process is dominated by the dumbbell orientation rather than the velocity. Inset shows the distribution of the orientation angles ϕ for successful and unsuccessful formation of tetramers. (**H** and **I**) Spinning angular velocities ω for (H) tetramers and (I) hexamers as function of *E*. Inset in (H) is the evolution of the orientation angle $\theta_{\hat{n}}$ as the tetramer spins. Inset in (I) shows the mean angular displacement 〈Δθ(*t*)^2^〉 of a spinning hexamer. *E* = 3.1 V μm^−1^.

In some respects, the single-dumbbell behavior that we observe is similar to that seen in the study of Zhang *et al.* (*43*), who studied the collective behavior of pear-shaped Quincke rollers. In particular, they also see a nonmonotonic persistence length, as a function of field strength [figure 3D in (*43*)] is compatible with the nonmonotonic radius of curvature shown in Fig. 2A (inset), although the radii of the orbital motion is very much larger in our case. However, the sequence of states that they observed seems opposite to the single-dumbbell behavior here, as they see spinners and vortices as a function of decreasing field strength, while we see spinners and DO and OO motions as a function of increasing field strength. Given that the particles in (*43*) are also Quincke rollers, we presume that the difference in behavior is related to the different shape. In particular, the aspect ratio of the particles in the work of Zhang *et al.* (*43*) is much closer to that of a sphere than is the case for the dumbbells that we consider here. We return to this point in the conclusion.

### Dumbbell collisions: Hierarchy of “excited states”

Having a suspension of dumbbells performing DO motion, e.g., at *E* ∈ {2.5,3.5} V μm^−1^, we observe collisions between dumbbells that lead to the formation of tetramers and more complex hexamers. Figure 3A shows the sequential formation of these excited states. First, isolated dumbbells collide and interact. If the collision is successful in terms of alignment, then the result is an excited bound state in the form of spinning tetramer of rhomboidal shape (see Fig. 3B and movie S2). We term this state “excited” due to the increased frequency of rotation (see Fig. 3H, inset) with respect to unbound dumbbells at the same field strength. By analogy with atomic systems, we term these states of a fixed number of bound particles active colloidal molecules. We argue that this “excited state” is due to the dumbbells colliding and being unable to move past one another following the collision. That is, the particle geometry enables dynamical self-trapping somewhat reminiscent of motility-induced phase separation (*5*), which, here, results in a bound state.

For colliding dumbbells, we measure the angle ϕ made between the orientations ${\hat{n}}_{ij}^{⊥}$ and velocities **v***_ij_* before collision and tetramer formation. Figure 3E shows the distributions of the $\phi_{\hat{n},v}$ angles. While the displacements exhibit a broader distribution, the successful formation of tetramers is governed by the orientation of the dumbbell trajectories. That is to say, the formation of tetramers is achieved by dumbbells on a collision course and such that their orientation angle ϕ → π and the displacements **v***_i_* + **v***_j_* = 0. For spherical and pear-shaped rollers, alignment from hydrodynamic interactions leads to the formation of collective phases, e.g., phased-locked trajectories. On the other hand, the motion of the dumbbell rollers described here is dominated by fast rotations that, in the event of a collision, frustrate alignment interactions and orbital trajectories.

We compare successful tetramer formation against other dumbbell collisions, confirming the strong dependence on orientation (see inset in Fig. 3E). If unperturbed, then tetramers spin at a constant angular velocity ω and without notable displacement of the center of mass Δ**r**. The rotation results from the torque as the center of propulsion from each dumbbell is not aligned with the center of mass of the tetramer (Fig. 3B). Otherwise, any substantial change in the orientation ${\hat{n}}_{⊥}$ promotes tetramer breaking and reversal to the circular motion of dumbbells. Figure 3H depicts the spinning speed ω of tetramers as a function of *E*. We observe increasing ω with the field as a result of the enhanced self-propulsion *v* (Fig. 2D).

We presume that the mechanism for the coupling is related to hydrodynamic interactions between the rotating dumbbells as indicated in Fig. 3D. It is possible that parallels may be drawn with predictions for hydrodynamically bound states in other active systems (*30*, *31*), for example, the volvox algae in experiments (*32*). Note that we only observe the formation of tetramers in the DO state. We presume that this is because the translational motion of spinning dumbbells is rather slow and so they do not collide; in the OO state, the dumbbells tend to follow the same trajectory, so their chance for collision is reduced also.

### Hexamers: Unstable excited states

In a more complex scenario, spinning hexamers form because of the self-trapping of three dumbbells. For this, an additional dumbbell collides with a preexisting tetramer. A triangular hexamer results from the local rearrangement of dumbbells, as represented in Fig. 3, (B and C) (see also movie S4). Similar to tetramers, it is likely that the process is governed by the dumbbell orientation ${\hat{n}}_{⊥}$. In Fig. 3F, we show an experimental formation sequence of a hexamer, where the orientations ${\hat{n}}_{i,j,k}$ for each dumbbell are highlighted. We find a few spinning hexamers using the same values of *E* as for the tetramers. This is given by the trajectories of individual dumbbells (see Fig. 2, B and E). The spinning speed shows a linear increase with *E*, suggesting a stronger coupling of the individual self-propulsion speeds *v* (Fig. 3I). In contrast to tetramers, the breaking of hexamers shows no reversion, as any deviation of the individual orientation $\hat{n}$ leads to the segregation of the constituent dumbbells (movie S3). Thus, the hexamers are much shorter-lived than the tetramers. The break-up of the hexamer in movie S3 underlines the complex hydrodynamic couplings in colloidal system under dc fields.

We emphasize that the collision processes here are different to the phoretic and hydrodynamic interactions of Janus particles (*1*) and our Quincke rollers. The formation of tetramers and hexamers is due to dynamical self-trapping, akin to systems displaying motility-induced phase separation (*4*). Therefore, the active pathway of hierarchical states is distinctive from those observed in systems with induced interactions (*45*).

### Trimers: Flipping on a honeycomb lattice

**Fig. 4.**
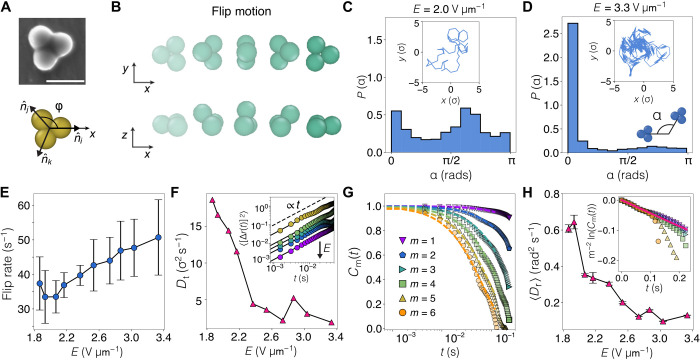
Quincke trimers. (**A**) Top: SEM micrograph of a Quincke trimer. Scale bar, 5 μm. Bottom: Trimer body frame. The orientation $\hat{n}$ of each vertex is given by an angle φ formed with respect to a reference axis and each vertex position. (**B**) Schematic representation of the flip motion performed by active trimers. Every jump corresponds to a leapfrogging mechanism of one vertex over the opposite side of the trimer, which takes the trimer out of the plane close to the substrate. (**C**) Distribution of flip angles α for a trajectory *E* ≈ 2 V μm^−1^, shown at the inset. (**D**) A trimer trajectory dominated by flips at *E* ≈ 3.33 V μm^−1^ (inset) shows a strong distribution of α → 0. (**E**) Flip rate as function of the electric field strength *E*. (**F**) Effective translational diffusion coefficients *D*_t_ obtained from filtered trajectories. Inset shows diffusive MSDs. Arrow indicates increase in *E*. (**G**) Reorientational time correlation functions for six different ranks *m* as defined in the main text. Symbols are obtained from experimental trajectories at *E* ≈ 2 V μm^−1^, and dashed lines are fittings from Eq. 7. Inset in (H) displays the collapsed scaled functions for the same data shown in (G). Solid line is a fitting using the mean rotational diffusion coefficient *D*_r_ extracted from the fits in (G). (**H**) Effective rotational diffusion coefficients *D*_r_ versus the applied electric field strengths.

We now proceed to describe the active motion of trimers, which are rigid assemblies of three particles (Fig. 4A). Quite unlike monomers or dumbbells, a trimer cannot simply rotate in response to the applied field. Instead, they undergo a flipping motion, where the trimer lies parallel to the substrate and from time to time flips rapidly about one side. Hence, we identify a class of Quincke flippers. This consists of a jump performed by one vertex over to the opposite side, as represented in Fig. 4B (see movie S5). Such a jump effectively instantaneously rotates the orientation by π about an axis parallel to the triangle side and displaces the center of mass **r** by a distance ℓ perpendicular to this axis. Assuming nonslip conditions, ℓ ≈ 0.7σ. In between flips, there is continuous (possibly diffusive) evolution of position and orientation. In the absence of evidence to the contrary, it seems reasonable to assume that these two types of motion occur independently.

The orientation of the trimer is specified by three Euler angles (φ, θ, ψ). The first angle, φ, is between the body-fixed and space-fixed *x* axes; we take the body-fixed *x* axis to point from the trimer center toward a vertex (see Fig. 4A). The second angle, θ, is a rotation about the body-fixed *x* axis. This takes values 0 and π in the unflipped and flipped state, respectively, and, within the instantaneous flip approximation, these are the only values of interest. The third angle, ψ, may be taken to be zero. Assuming that the dynamics do not depend on the flip state, we may focus on the angle φ alone.

For a trimer with particle centers in an equilateral geometry, a flip may be represented as φ → φ + Δφ, **r** → **r** + Δ**r**, where Δφ = ± π/3, π corresponding to the three possible flip directions, and

Δr=ℓ[cos(φ−Δφ),sin(φ−Δφ)] (5)

In the absence of motion between flips, the center **r** of each trimer would explore the vertices of a two-dimensional honeycomb lattice. Successive flips may or may not be correlated, regarding the time intervals between flips and/or the choice of successive flip directions. The simplest model for the motion between flips is that the trimers translate and rotate diffusively, obeying

$$\dot{r}=(\dot{x},\dot{y})=\sqrt{2D_{t}}(\zeta_{x},\zeta_{y})\;\mathrm{and}\;\dot{\varphi }=\sqrt{2D_{r}}\zeta_{\varphi }$$ (6)

where ζ*_x_*, ζ*_y_*, and ζ_φ_ are independent delta-correlated stationary Gaussian processes with zero mean and *D*_t_ and *D*_r_ are the translational and rotational diffusion coefficients. An active (velocity) contribution might be added to these equations, but such a term would imply some breaking of triangular symmetry.

Figure 4 (C and D) displays experimental trajectories of a trimer performing an essentially random walk at different activities. In contrast to active spheres and dumbbells, trimers show reduced displacement Δ**r** due to the symmetry in jumps. For the range of field strengths applied here, i.e., *E* ∈ {1.8,3.4} V μm^−1^, we find that the motion of trimers is dominated by flips. To characterize any correlation of the flips, we define an angle α made by two successive displacements of the center of mass (see the diagram in Fig. 4D). Having uncorrelated flips without rotation in between, the angle made by Δ**r** takes possible values +π/3, −π/3, or π occurring with equal probability. For α (as defined in Fig. 4D), the angles for the three previous cases are 2π/3, 2π/3 (again), and 0. Then, we find a reasonable approximation at lower field strength, as shown in Fig. 4C, albeit with a larger peak at α ≈ 2π/3. Upon increasing *E*, we observe weakening of the bimodal nature of the distribution as the distribution of α shifts to 0, showing an enhanced anisotropic motion, as displayed in Fig. 4D. That is, upon increasing the field strength, the trimers exhibit a greater tendency to flip forward and back. In addition, the increase in *E* yields an increasing flip rate, as shown in Fig. 4E. The increasing correlation of flips might be given by any small asymmetry in the shape, i.e., spheres of different sizes, which, together with the increased field, result in linear regions of the trajectory (see inset in Fig. 4D).

With the above observations in mind, it is possible to devise a dynamical model of the trimer, based on continuous (possibly diffusive) motion, punctuated by instantaneous flips (possibly incorporating the correlations just discussed), to compare with experiments (*55*–*64*). This model is discussed further in the Supplementary Materials (51). Here, we focus on isolating the continuous motion and determining whether it is diffusive. Using the facts that the flips are rapid and produce displacements Δ**r** and Δφ that approximately satisfy Eq. 6, it is possible to remove the effects of the flips from the experimentally observed trajectories, leaving just the continuous evolution of **r**(*t*) and φ(*t*). We refer to these as filtered trajectories. We emphasize that this is an artificial procedure, only likely to be successful if the two types of motion are sufficiently independent.

MSDs of filtered trajectories obtained at different field strengths *E* are shown in the inset of Fig. 4F. The curves can be fitted by 〈Δ*r*(*t*)^2^〉 = 4*D*_t_*t*, and values for the effective diffusion coefficient *D*_t_ are shown as a function of *E* in Fig. 4F. At first sight, it may seem unexpected that the effective diffusion coefficient decreases as a function of field strength, while the activity increases. We believe that this is due to an increased tendency to flip forward and back, as indicated in Fig. 4D. This then suppresses the displacement of the center of mass of the trimer, leading to the reduction in the effective diffusion constant. The filtering that we carry out has no impact on this behavior.

Reorientation in the plane is analyzed by means of time correlation functions of φ and again extracted from the filtered trajectories

$$C_{m}(t)=\langle \cos m\Delta \varphi (t)\rangle =\exp(-m^{2}D_{r}t)$$ (7)

where Δφ(*t*) = φ(*t*) − φ(0), the change in angle due to nonflip motion only, *m* is the rank, and the last expression is expected for pure rotational diffusion with coefficient *D*_r_ (*56*). In Fig. 4G, we show results for *m* ≤ 6 at one value of *E*. In the inset of Fig. 4H, the same data collapse onto a single curve *m*^−2^ ln *C*_m_(*t*) versus *t*, allowing an estimate of *D*_r_. Rotational diffusion coefficients *D*_r_ as a function of *E* are shown in Fig. 4H. Translational and rotational diffusion seems to satisfactorily describe the motion between flips. We find decay of both *D*_t_ and *D*_r_ as we increase *E*. It is important to recognize that perfect separation of flips and diffusion may be impractical (in reality, there is a distribution of flip distances and directions, and the trimers are not perfectly equilateral triangles). Hence, the measured “diffusion” coefficients may include residual contributions from the flips. If this is the case, then the decrease in *D*_t_ and *D*_r_ with increasing *E* might be connected with the increased importance of the α = 0 peak, i.e., correlated forward and backward flips, at higher *E*, indicated in Fig. 4D.

## DISCUSSION

In summary, we have investigated active motion of dumbbells and trimers powered by Quincke rotation. The orbital motion and the flipping behavior of these nonspherical particles are markedly different from that observed in rolling colloids. For both cases, the behavior is controlled by the applied electric field. The motion of dumbbells observed experimentally at intermediate activity is in agreement with the theoretical description of a circle swimmer (*49*).

In the case of single Quincke dumbbells, as a function of increasing field strength, we observe spinners with no translational active motion, followed by states of DO and then OO motions. This motion transforms into the emergence of spinning tetramers and hexamers as dumbbells collide with each other. This corresponds to the formation of excited bound states arising from the hydrodynamic coupling of dumbbells. The persistence length inherent in the motion of Quincke Rollers leads to a dependence on the trajectories of the incoming dumbbells and particularly on the collision angle ϕ (Fig. 3A). Such a dependence is absent from the overdamped dynamics of passive colloidal systems, and the exotic excited sates that we find are reminiscent of long-lived complexes formed by collisions between molecules but at the colloidal lengthscale and, of course, with classical interactions (*29*). It is possible that at higher concentrations of dimers, a collective demixing reminiscent of motility-induced phase separation (MIPS) might be observed, similar to that seen for some other anisotropic colloids (*42*), although a full understanding would likely need to include the hydrodynamic interactions between dumbbells and the spinning tetramer and hexamer excited states. Last, we have shown the flip behavior of trimers, which is described by means of a jump-diffusion model. We have implemented a minimal model and leave the detailed mechanism for the future. Under our assumptions, the model reproduces the experiments rather accurately.

Our findings contrast with collective behavior in a system of pear-shaped Quincke rollers in (*43*), who found a phase of particles also exhibiting orbital motion. However, they do not appear to find anything similar to the spinning state that we find at lower field strength. Furthermore, they do not report coupling of pairs of particles, so that system seems not to exhibit behavior similar to the excited bound state of tetramers and hexamers that we observe (Fig. 3). We presume that the reason for this is the geometry of the pear-shaped particles whose aspect ratio is closer to that of a sphere and, moreover, does not appear to enable locking of colliding particles. In addition, for geometric reasons, the hopping of the trimers reported here is not found in the pear-shaped particles. The disk-shaped particles in (*42*) seem to be able to transiently form bound states reminiscent of the tetramers that we observe. This is consistent with their relatively large aspect ratio. However, they do not seem to interlock, and perhaps as a consequence, these states are shorter lived than the tetramers. The role of particle shape in influencing interactions and assembly in Quincke rollers and other active particles is clearly an intriguing topic for the future.

The experiments that are now possible with this system may be beneficial for the investigation of different types of motion as encountered in nature, as well as for the design of nonequilibrium self-assembly routes, and to provide a readily observable classical analog of collisions and excited states in molecules. In particular, our work opens the way to active supracolloidal chemistry. Other geometries of cluster in addition to dumbbells and trimers could be explored (*23*). Here, we have considered the dilute limit with pairwise collisions and interactions. A particularly interesting avenue to explore would be higher concentration, which has been achieved in the case of (passive) colloidal dumbbells (*65*) and anisotropic active colloids with other geometries (*42*, *43*), with which predictions from computer simulation might be explored (*39*). Inclusion of active control (*17*) opens even more exciting possibilities.

## MATERIALS AND METHODS

Colloidal molecules are prepared as follows. We use polystyrene beads (Fluoro-Max, Thermo Fisher Scientific) of size σ = 3.1 μm and a polydispersity of 5% as determined by scanning electron microscopy (SEM). The initial suspension is aqueous. Colloids are repeatedly washed with a 0.15 M solution of dioctyl sodium sulfosuccinate (AOT) surfactant in hexadecane. In the absence of a steric stabilizing layer, colloidal clusters form because of van der Waals attractions. We obtain a mixture of clusters as the aqueous solvent is replaced by the low polar solution. Centrifugation is used to separate small clusters, i.e., dumbbells and trimers, from the rest of the suspension.

For the experiments, a dilute mixture of clusters is loaded into a sample cell fabricated with conductive indium tin oxide–coated glass slides (ITOSOL-12, Solemns). Two slides are separated by a 30-μm-thick spacer made of optical glue and larger beads. An amplified (Trek 606E-6) dc electric field *E* is applied to the suspension to observe the Quincke electrorotation of colloids (*3*). Image sequences are obtained at 660 frames/s using bright-field microscopy (Leica DMI 300B) and a digital camera (Basler ACE). The monomers, dumbbells, and trimers appear to be colloidally stable on the time scale of the experiment. That is to say, we saw no sign of aggregation nor any change in the populations of any of the three species.
