# Supplementary material for: Dynamics and interactions of Quincke roller clusters: From orbits and flips to excited states
Source: Sci Adv. 2023 May 17;9(20):eadf5144. doi: 10.1126/sciadv.adf5144 (PMC10191443; doi:10.1126/sciadv.adf5144)
Supplement: Supplementary file 1 — Figs. S1 and S2 Legends for movies S1 to S5 [file sciadv.adf5144_sm.pdf]

Science Advances
AAAS

Supplementary Materials for

Dynamics and interactions of Quincke roller clusters: From orbits and flips to excited states

Abraham Mauleon-Amieva *et al.*

Corresponding author: C. Patrick Royall, paddy.royall@espci.psl.eu

Sci. Adv. **9**, eadf5144 (2023)
DOI: 10.1126/sciadv.adf5144

The PDF file includes:

Figs. S1 and S2
Legends for movies S1 to S5

Other Supplementary Material for this manuscript includes the following:

Movies S1 to S5

A HYDRODYNAMIC SINGULARITY MODEL OF DUMBBELLS

The dumbbell motion is due to electrokinetic flows generated by the interaction with the applied electric field and the colloids which leads to an attraction between the colloids (see Refs. [38,52] in the main text). The in-plane dumbbell rotation arises as soon as this effective attractive interaction is not parallel to the line between the colloid centres. We show this below by calculating the hydrodynamic flow fields thus generated.

We consider the colloids resting on a flat surface in an incompressible fluid of viscosity η. In the vanishing Re limit of creeping hydrodynamic flows, the far field behaviour of this system is well described using hydrodynamic singularities due to point forces near a surface, using the method of images.

Given a pair of spherical colloids of radius $\simeq a$ on a flat surface with centres at positions $\mathbf{r}_1 = (x_1, y_1, a)$ $\mathbf{r}_2 = (x_2, y_2, a)$, separated by the vector $\mathbf{r}_2 - \mathbf{r}_1 = \mathbf{d}$ and with attractive forces $\mathbf{f}_1 = -\mathbf{f}_2 = (f_x, f_y, 0)$ which are not parallel to $\mathbf{d}$, we can write the equations of motion for the fluid velocity $\mathbf{v}(\mathbf{r})$ and pressure $p(\mathbf{r})$ where $\mathbf{r} = (x, y, z)$ as

$$\eta\nabla^2\mathbf{v} - \boldsymbol{\nabla} p = \sum_{i=1}^{2} \mathbf{f}_i \delta(\mathbf{r} - \mathbf{r}_i) \quad , \quad \boldsymbol{\nabla}\cdot\mathbf{v} = 0 \quad , \tag{1}$$

with boundary conditions $\lim_{|\mathbf{r}|\to\infty} \mathbf{v}(\mathbf{r}) = 0, \lim_{|\mathbf{r}|\to\infty} p(\mathbf{r}) = 0$ and $\lim_{z\to 0} \mathbf{v}(\mathbf{r}) = 0$.

The flow field generated is then given by Ref. [53] in the main text,

$$\mathbf{v}(\mathbf{r}) = \sum_{i=1,2} \left[\frac{1}{8\pi\eta} \left(\mathbf{H}(\mathbf{r} - \mathbf{r}_i) + \mathbf{G}(\mathbf{r} - \mathbf{R}_i) \right) \cdot \mathbf{f}_i \right] \tag{2}$$

where $\mathbf{R}_i = (x_i, y_i, -a)$ are the positions of the images and the tensors $\mathbf{H}, \mathbf{G}$ are given by

$$H_{\alpha\beta}(\mathbf{r}) = \frac{1}{r^3} \left(r^2 \delta_{\alpha\beta} + r_\alpha r_\beta \right) \tag{3}$$

$$G_{\alpha\beta}(\mathbf{r}) = -H_{\alpha\beta}(\mathbf{r}) + (1 - 2\delta_{z\beta}) \frac{\partial}{\partial r_\beta} \left(\frac{2a^2 r_\alpha}{r^3} - 2aH_{\alpha z}(\mathbf{r}) \right) \quad . \tag{4}$$

The full 3D flow field generated by Eqn. 2 is given in Fig. 1(e) and the 2D projection on the plane $z = a$ is given in Fig. 1(d) in the main text.

Finally, we find the dumbbell spins with an angular speed

$$\Omega \simeq \frac{|\mathbf{d}|^{-1} |\mathbf{f}_\perp|}{\zeta} \tag{5}$$

where $\mathbf{f}_\perp = \mathbf{f}_1 - \left(\mathbf{f}_1 \cdot \hat{\mathbf{d}} \right) \hat{\mathbf{d}}$, $\hat{\mathbf{d}} = \mathbf{d}/|\mathbf{d}|$ and ζ is the Stokes drag of the colloid near the surface.

Once we go through the Quincke threshold and the colloids begin to roll, we must add additional singularities (Stokeslet + image) at the dumbbell centre of mass to the flow field.

JUMP–DIFFUSION MODEL

To assist in interpreting the trajectories of the Quincke trimers, we consider the idealized model of a rigid equilateral triangle undergoing translational and rotational diffusion in the plane, punctuated by flips. Each flip consists of an instantaneous rotation of the triangle by an angle π about one of the edges, chosen at random. The model makes no attempt to represent the physical driving forces behind the motion, and in fact is not an “active matter” model as such.

We denote the particle centre of mass $\mathbf{r} = (x, y)$, and define the orientation by Euler angles (φ, θ, ψ), in the classic zxz convention. This means that the first angle φ, of rotation about the $\mathbf{z}$-axis perpendicular to the plane, is the angle between the space-fixed and body-fixed $\mathbf{x}$-axes. The second angle θ, of rotation about the body-fixed $\mathbf{x}$-axis, takes values $\theta = 0$ (unflipped), or $\theta = \pi$ (flipped). The final angle is always $\psi = 0$.

Between flips, simple diffusion in the absence of external forces, over a time interval Δt, evolves $\mathbf{r}$ and φ as follows:

$$\Delta x = \sqrt{2D_\mathrm{t}\Delta t}\,\mathcal{N}_x,\ \Delta y = \sqrt{2D_\mathrm{t}\Delta t}\,\mathcal{N}_y,\ \Delta\varphi = \sqrt{2D_\mathrm{r}\Delta t}\,\mathcal{N}_\varphi,$$

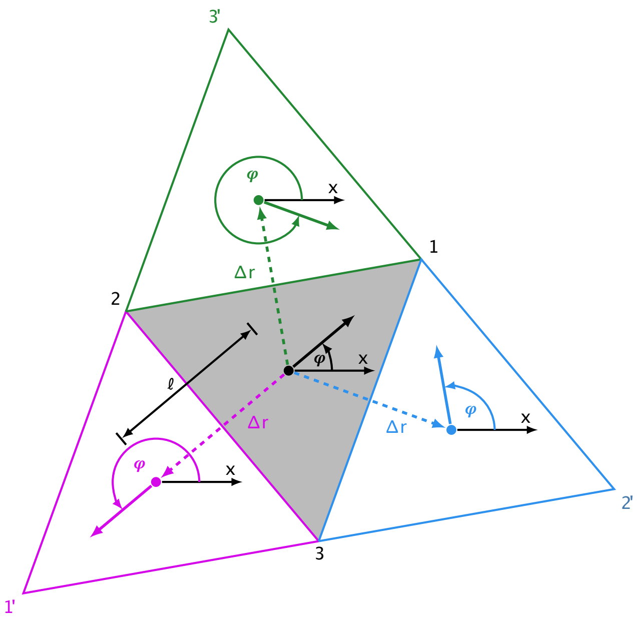

FIG. S1. Geometry of triangle flips. The original triangle is shaded. The vertices are numbered anticlockwise, assuming an unflipped state. The coloured triangles are the three possible flipped configurations. The flipping vertex number is identified with a prime. The arrowed lines indicate the space-fixed and body-fixed **x**-axes, and the angles φ are shown. The body-fixed **x**-axis always points from the centre of the triangle towards vertex 1. In the unflipped (flipped) state, the body-fixed **z**-axis points upward (downward) out of the plane. In the jump–diffusion model, we just follow the reorientation of the **x**-axis. The dashed arrowed lines indicate the displacements of the centre in each case.

where D_t and D_r are the translational and rotational diffusion coefficients, and the $\mathcal{N}_\alpha$ are independent random numbers chosen from the normal distribution (Gaussian with zero mean and unit variance).

The flips discontinuously change $\mathbf{r}$ and both φ and θ. The effect on θ is always $\theta \to \pi - \theta$. The dynamics does not depend on θ, but it is needed to track, for example, the triangle vertex positions. The dynamics described above may be used as the basis of a simple simulation program to generate trajectories, for analysis and comparison with experiment, or (with some further assumptions) to produce analytical predictions. We describe some of these in the following.

If we focus on the reorientation of the space-fixed **x**-axis, we may restrict our interest to $\mathbf{r}$ and φ alone, and represent the dynamics as a *jump–diffusion* process in the plane. This is an oversimplification, since the flip is not equivalent to a simple rotation in the plane, but time correlation functions based on $\mathbf{r}$ and φ should still provide a useful point of comparison with the same quantities calculated from experimental trajectories. Referring to Fig. S1, it is straightforward to show that the flips generate three possible changes, which we label $\alpha = 1, 2, 3$:

$$\varphi \to \varphi + \Delta\varphi_\alpha, \qquad \Delta\varphi_\alpha = (2\alpha - 1)\pi/3, \tag{6a}$$

$$\mathbf{r} \to \mathbf{r} + \Delta\mathbf{r}_\alpha, \qquad \Delta\mathbf{r}_\alpha = \ell\big(\cos(\varphi - \Delta\varphi_\alpha), \sin(\varphi - \Delta\varphi_\alpha)\big). \tag{6b}$$

The jump distance is ℓ, and the side of the triangle is $\sqrt{3}\ell$.

In the following, we assume that the jumps are independent uncorrelated events, obeying the Poisson distribution

$$P(n_\alpha, t) = \frac{(\kappa_\alpha t)^{n_\alpha}}{n_\alpha!} \mathrm{e}^{-\kappa_\alpha t}. \tag{7}$$

Here, $P(n_\alpha, t)$ is the probability of exactly n_α jumps of type $\alpha = 1, 2, 3$ (corresponding to the three cases described above) during time interval t, and κ_α is the corresponding jump rate. Moreover, we assume that each type of jump is equally likely, so $\kappa_\alpha = \kappa/3$ where $\kappa = 1/\tau$ is the overall jump rate and τ the average interval between jumps of any type.

Reorientation is described in the standard way (see Refs. [56-59] in the main text), via time correlation functions of various ranks m:

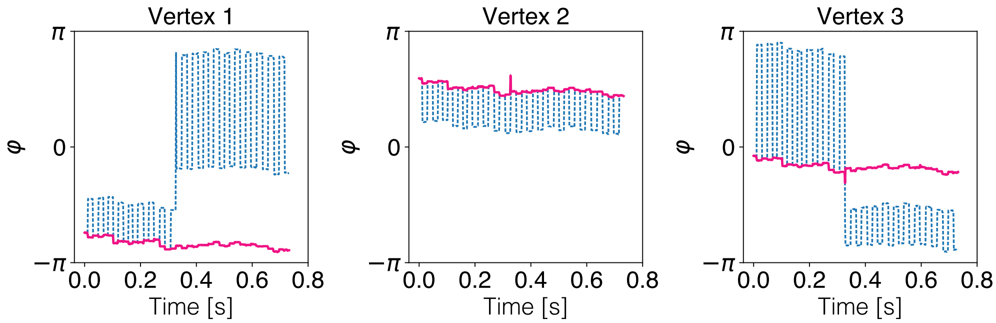

FIG. S2. Filtered trajectories of trimers. Dashed lines indicate the evolution of the orientation φ for each vertex when flips occur. Each flip discontinuously evolves the orientation φ of each vertex by π, $\pi/3$, or $-\pi/3$ respectively. Solid lines represent the change of φ when the effect of flips is removed from the original trajectories.

$$C_m(t) = \big\langle \cos m\Delta\varphi(t)\big\rangle = \Re\big\langle \mathrm{e}^{-\mathrm{i}m\Delta\varphi(t)}\big\rangle = \Re\big\langle \mathrm{e}^{-\mathrm{i}m\varphi(t)}\,\mathrm{e}^{\mathrm{i}m\varphi(0)}\big\rangle$$

where $\langle\cdots\rangle$ denotes a statistical average, and $\Re$ indicates the real part. In an isotropic environment, these functions are expected to decay to zero for all m. In the rotational diffusion limit

$$C_m(t) = \mathrm{e}^{-m^2 D_\mathrm{r} t}, \tag{8a}$$

$$\Rightarrow \quad m^{-2}\ln C_m(t) = -D_\mathrm{r} t \tag{8b}$$

where D_r is the rotational diffusion coefficient. Eqn. (8b) may be used to collapse the results for several values of m onto a single plot from which the value of D_r may be inferred. For the rotational jump model with uncorrelated jumps obeying Eqn. (7), it is possible to derive the result (see Ref. [60] in the main text),

$$C_m(t) = \exp\left[-\sum_\alpha \kappa_\alpha t\big(1-\cos(m\Delta\varphi_\alpha)\big)\right]\cos\left[\sum_\alpha \kappa_\alpha t\sin(m\Delta\varphi_\alpha)\right]. \tag{9}$$

For the three $\Delta\varphi_\alpha$ values listed in Eqn. (6a), occurring at equal rates κ_α, each $C_m(t)$ decays exponentially in time, at a rate depending on m: $C_m(t) = \exp(-\gamma_m t/\tau)$, where τ is the interval between jumps. The first six decay rates are

$$\gamma_1 = \gamma_2 = \gamma_4 = \gamma_5 = 1, \quad \gamma_3 = 2, \quad \gamma_6 = 0.$$

The jump rotations do not affect the $m = 6$ function at all, since the jump angles are integer multiples of $\pi/3$. This non-monotonic dependence of decay rate on m is in contrast to the rotational diffusion result. For our model, in which both diffusional and jump motions are occurring, it is easy to show that the effects on the decay rate are additive: $C_m(t) = \exp\big[-(\gamma_m/\tau + m^2 D_\mathrm{r})t\big]$.

The experimental results described in the main text suggest some correlation between trimer flips, and this would affect the rotational correlation functions just defined. In principle, it is possible to extend the above model to account for this (Ref. [60] in the main text), but more quantitative experimental data regarding the nature of the correlations would be needed for comparison. Instead, in the main text, we attempt to filter out the effects of the flips (see Fig. S2) leaving just a residual rotational diffusion, which is confirmed through Eqn. (8b).

Turning to the translational motion, the mean-squared displacement for our model is easily calculated as a function of time, but is not especially revealing. The lack of correlation between successive jumps leads to

$$\big\langle|\Delta\mathbf{r}(t)|^2\big\rangle = (4D_\mathrm{t} + \ell^2/\tau)t \tag{10}$$

for all t, irrespective of the value of D_r. It is possible to consider non-Poisson distributions of jump intervals (see Ref. [61] in the main text) but, for the reasons discussed above, more experimental details would be needed before attempting a comparison. Instead, once more, analysis of filtered trajectories enables an estimate to be made of D_t. However, because of the form of Eqn. (10), unlike the rotational case, there is no in-built check from this kind of

measurement that the contribution of jumps has been completely filtered out. The single-particle ('self') intermediate scattering function

$$F_{\rm s}(\mathbf{q},t) = \Re\left\langle {\rm e}^{-{\rm i}\mathbf{q}\cdot\Delta\mathbf{r}(t)}\right\rangle = \Re\left\langle {\rm e}^{-{\rm i}\mathbf{q}\cdot\mathbf{r}(t)}\,{\rm e}^{+{\rm i}\mathbf{q}\cdot\mathbf{r}(0)}\right\rangle,$$

where $\mathbf{q}$ is the wave-vector, would provide a clearer way of discriminating between the diffusional and jump motion, and perhaps quantifying both. As in the rotational case, there is insufficient information from the experimental trajectories to properly compare with theory or simulation, and so we do not pursue this in the main text. For completeness, however, here are the relevant theoretical results, once more assuming that the intervals between jumps obey Eqn. (7).

In the limit of high rotational diffusion coefficient $D_{\rm r}$, it is reasonable to assume that the directions of successive jumps are uncorrelated. In this case, a simple isotropic jump model (Ref. [62] in the main text) would predict, in 2D,

$$F_{\rm s}(\mathbf{q},t) = \exp\bigl(f(\mathbf{q})t/\tau\bigr) \quad \text{where} \quad f(\mathbf{q}) = J_0(q\ell) - 1.$$

Here $J_0(x)$ is the Bessel function of the first kind, τ the interval between jumps, and ℓ the jump distance. For small $q\ell$, an expansion gives $f(q) = -\frac{1}{4}q^2\ell^2$, and hence a diffusive form $F_{\rm s}(\mathbf{q},t) = \exp(-\frac{1}{4}q^2\ell^2 t/\tau)$. Incorporating the effects of translational diffusion between the jumps will simply multiply $F_{\rm s}(\mathbf{q},t)$ by a factor $\exp(-D_{\rm t}q^2t)$. Examining $F_{\rm s}(\mathbf{q},t)$ at high values of $q\ell$ would enable the jump and diffusional contributions to be separated.

In the limit of vanishing $D_{\rm r}$, the jumps explore the honeycomb (hexagonal) lattice. This is a non-Bravais lattice, having two inequivalent types of site, A and B, and the jumps connect vertices belonging to the two different sublattices. The model has been well analyzed (see for example Refs. [63-65] in the main text) and we only give a brief summary of the result here. Let $\{\mathbf{a}_\alpha\}$, $\alpha = 1,2,3$, be the set of jump vectors, taking the triangle from a site on sublattice A to a nearest-neighbour site on sublattice B. These are equal to the $\Delta\mathbf{r}_\alpha$ vectors of Eqn. (6b) in the reference orientation $\varphi = 0$, and of course they are all of length ℓ. The complementary set of jumps from B to A is given by $\{-\mathbf{a}_\alpha\}$. It is convenient to define an amplitude X (real, non-negative) and a phase χ (real) as follows

$$\frac{1}{3}\sum_\alpha {\rm e}^{{\rm i}\mathbf{q}\cdot\mathbf{a}_\alpha} \equiv X{\rm e}^{{\rm i}\chi}.$$

Assuming equal jump rates, for any wave-vector $\mathbf{q}$, the self intermediate scattering function may be expressed as a sum of two exponential decays in time

$$F_{\rm s}(\mathbf{q},t) = c_+(\mathbf{q})\exp\bigl(f_+(\mathbf{q})t/\tau\bigr) + c_-(\mathbf{q})\exp\bigl(f_-(\mathbf{q})t/\tau\bigr), \tag{11}$$

where $f_\pm = \pm X - 1$, $c_\pm = \frac{1}{2}\bigl(1 \pm \cos\chi\bigr)$. It is easy to see that $0 \le X \le 1$, guaranteeing that $f_\pm \le 0$. At low $q\ell$ it can be shown that $X = 1 - \frac{1}{4}q^2\ell^2$ and $\chi = 0$, so $c_- = 0$, c_+=1, $f_+ = -\frac{1}{4}q^2\ell^2$, and $F_{\rm s}(\mathbf{q},t) = \exp(-\frac{1}{4}q^2\ell^2t/\tau)$ as expected. As before, incorporating the effects of translational diffusion between the jumps introduces a factor $\exp(-D_{\rm t}q^2t)$. Away from the low-$q\ell$ limit, Eqn. (11) varies significantly with both magnitude and direction of $\mathbf{q}$, and therefore allows one to distinguish between jump and diffusive contributions. For comparison with experimental results at low, but non-vanishing, $D_{\rm r}$, it would make sense to average Eqn. (11) over all directions of $\mathbf{q}$ in the plane.

For intermediate values of $D_{\rm r}$ we have not attempted to derive an analytical form of $F_{\rm s}(\mathbf{q},t)$. However, we have implemented a simple, single-particle, simulation program, for the case of Poisson-distributed independent jumps, and this provides very accurate numerical results. This approach should also be fruitful for correlated jumps, in the event that further experiments on the trimers provide sufficient information about the nature of the correlations.

DETAILS OF SUPPLEMENTARY MOVIES

Movie 1. Left panel: Spinning dumbbells appear at low field strengths. Middle and lefts panels: Quincke dumbbells exhibit circular and orbital-like motion with increasing field amplitude E. Scale bar is 50 μm. Movie recorded at 180 fps and played at 30 fps.

Movie 2. Collisions between dumbbells lead to spinning tetramers. The self-trapping mechanism is dependent on the dumbbell orientation, and any deviation leads to tetramer break off. Field amplitude $E = 2.9V\,\mu m^{-1}$. Scale bar

is 10 μm. Movie recorded at 180 fps, and played at 17 fps.

Movie 3. Formation of a spinning hexamers from addition collisions between dumbbells. Similar to tetramer, any re–orientation promotes break off. Field amplitude $E = 3.13V\ \mu m^{-1}$. Scale bar is 10 μm. Movie recorded at 180 fps, and played at 17 fps.

Movie 4. Persistent spinning motion of a hexamer made of three dumbbells. Field amplitude $E = 3.13V\ \mu m^{-1}$. Scale bar is 10 μm. Movie recorded at 180 fps, and played at 17 fps.

Movie 5. The motion of Quincke flippers is characterised by consecutive jumps that evolve the position and orientation of trimers. Field amplitude $E = 3.3V\ \mu m^{-1}$. Scale bar is 10 μm. Movie recorded at 180 fps, and played at 17 fps.
